# Efgartigimod improved health-related quality of life in generalized myasthenia gravis: results from a randomized, double-blind, placebo-controlled, phase 3 study (ADAPT)

**DOI:** 10.1007/s00415-022-11517-w

**Published:** 2023-01-04

**Authors:** Francesco Saccà, Carolina Barnett, Tuan Vu, Stojan Peric, Glenn A. Phillips, Sihui Zhao, Cynthia Z. Qi, Deborah Gelinas, Silvia Chiroli, Jan J. G. M. Verschuuren

**Affiliations:** 1 Federico II University of Naples, Naples, Italy; 2 Prosserman Centre for Neuromuscular Diseases, Toronto General Hospital/UHN, Toronto, ON Canada; 3 University of South Florida Morsani College of Medicine, Tampa, FL USA; 4 University of Belgrade—Faculty of Medicine, University Clinical Center of Serbia—Neurology Clinic, Belgrade, Serbia; 5 argenx US, Inc., Boston, MA USA; 6 argenx Switzerland SA, Geneva, Switzerland; 7 University Medical Center, RC Leiden, Netherlands

**Keywords:** Generalized myasthenia gravis, gMG, Efgartigimod, Quality of life, HRQoL, Patient-reported outcomes

## Abstract

There are substantial disease and health-related quality-of-life (HRQoL) burdens for many patients with myasthenia gravis (MG), especially for those whose disease symptoms are not well controlled. HRQoL measures such as the Myasthenia Gravis Quality of Life 15-item revised (MG-QOL15r) and EuroQoL 5-Dimensions 5-Levels (EQ-5D-5L) are vital for evaluating the clinical benefit of therapeutic interventions in patients with MG, as they assess the burden of disease and the effectiveness of treatment, as perceived by patients. The phase 3 ADAPT study (NCT03669588) demonstrated that efgartigimod—a novel neonatal Fc receptor inhibitor—was well tolerated and that acetylcholine receptor antibody–positive (AChR-Ab+) participants who received efgartigimod had statistically significant improvements in MG-specific clinical scale scores. The ancillary data reported here, which cover an additional treatment cycle, show that these participants had similar significant improvements in HRQoL measures, the MG-QOL15r and EQ-5D-5L utility and visual analog scales, and that these improvements were maintained in the second treatment cycle. Positive effects on HRQoL were rapid, seen as early as the first week of treatment in both treatment cycles, and maintained for up to 4 weeks in the follow-up–only portion of treatment cycles. The pattern of improvements in HRQoL paralleled changes in immunoglobulin G level, and correlational analyses show that improvements were consistent across HRQoL measures and with clinical efficacy measures in the ADAPT study. The substantial and durable improvements in HRQoL end points in this study demonstrate the broader benefit of treatment with efgartigimod beyond relief of immediate signs and symptoms of gMG.

**Supplementary Information:**

The online version contains supplementary material available at 10.1007/s00415-022-11517-w.

## Introduction

Myasthenia gravis (MG) is a rare, chronic autoimmune disease characterized by debilitating and potentially life-threatening muscle weakness and fatigue [1]. Targeting of the neuromuscular junction by pathogenic immunoglobulin G (IgG) results in reduced neuromuscular transmission [2, 3]. There are substantial disease and quality-of-life (QoL) burdens for many patients with MG [2, 4]. Negative effects on multiple aspects of well-being, including physical, psychological, and social health in population cohort studies [5] and significantly reduced QoL in cross-sectional studies, have been reported [6].

Approximately 50% of patients with MG have mood disorders [5, 7]. Depression has been reported in about one-third of patients with MG [7] and is correlated with worse disease severity [8]. In fact, depression has predicted decreased QoL scores in psychological and physical domains [9]. Anxiety is also common in patients with MG and is a significant predictor of decreased health-related QoL (HRQoL) [7, 10]. Of note, HRQoL issues have been reported more often for women with MG, patients with lower income levels and concomitant diseases [11], and those with more-severe MG [5, 12]. The following predictors of worse HRQoL were found in 230 participants with MG in a study by Basta and colleagues: older age, lower education, more-severe MG, less social support, poor acceptance of the disease, and higher levels of anxiety and depression [10]. Another 10-year study showed that a significant number of participants with MG who had been in remission for up to 10 years still had reduced HRQoL [13].

Treatments for MG include acetylcholinesterase (AChE) inhibitors to control symptoms and immunosuppressive medications and thymectomy in individuals with acetylcholine receptor antibody–positive (AChR-Ab+) MG. Immunosuppressants used to treat MG include corticosteroids, nonsteroidal immunosuppressant therapies (NSISTs), and monoclonal antibodies such as rituximab, eculizumab, and ravulizumab [14–16]. Additionally, intravenous immunoglobulin and plasma exchange provide immunomodulation in disease-worsening episodes [15]. Nevertheless, some patients’ treatment needs remain unmet by these options.

Unsurprisingly, patients with MG whose disease symptoms are not controlled adequately with therapy or who have a high level of treatment side effects or burden report the lowest HRQoL [2]. In general, in patients with autoimmune disorders, systemic corticosteroids and long-term immunosuppressive treatment have been associated with lower HRQoL [17, 18]. Therapeutics for MG that carry a less-onerous side effect profile and are effective in improving disease signs and symptoms may help patients achieve better HRQoL. Efgartigimod alfa (hereafter, efgartigimod) is a novel antibody fragment and first-in-class neonatal Fc receptor (FcRn) antagonist [19], approved in December 2021 by the US Food and Drug Administration for AChR-Ab+ MG [20] and in Japan [19] for generalized MG (gMG). By blocking FcRn, efgartigimod reduces levels of circulating immunoglobulins, including pathogenic autoantibodies.

The phase 3 ADAPT study (NCT03669588) [21] supporting regulatory approvals of efgartigimod [20] investigated efficacy, safety, tolerability, impact on normal daily activities, and HRQoL in participants with gMG treated with efgartigimod. Efgartigimod was well tolerated, and efgartigimod-treated participants had statistically significant improvements in disease severity scores on HRQoL-related measures used to assess treatment efficacy [22].

The purpose of this secondary analysis is to report the HRQoL outcomes from participants with AChR-Ab+ gMG in the ADAPT study, across 2 cycles of treatment and follow-up. HRQoL measures used included the Myasthenia Gravis-Quality of Life 15-item revised (MG-QoL15r), which is an MG-specific measure, and the EuroQoL 5-Dimensions 5-Levels (EQ-5D-5L), including visual analog scale (VAS), which is a generic measure used across disease states.

## Methods

### Study design

ADAPT was a randomized, double-blind, placebo-controlled, multicenter, phase 3 study [22] conducted in 56 neuromuscular centers in North America, Europe, and Japan (15 countries total). Participants were randomly assigned in a 1:1 ratio to efgartigimod or placebo. Total study duration was up to 28 weeks, which included a 2-week screening period and a 26-week treatment period, consisting of up to three 8-week treatment cycles (TCs). In each TC, participants were administered 4 once-weekly intravenous (IV) infusions of 10 mg/kg efgartigimod or matching placebo (on days 1, 8, 15, and 22), then followed for the remainder of the TC without further treatment. Between TCs was an intertreatment cycle (ITC) of ≥ 5 weeks, the duration of which was based on each patient’s loss of treatment effect response, as indicated by predefined change in Myasthenia Gravis Activities of Daily Living (MG-ADL). This manuscript reports data from the first 2 TCs.

The ADAPT study was conducted according to the International Conference on Harmonisation Guideline for Good Clinical Practice, the principles of the Declaration of Helsinki, and other applicable local ethical and legal requirements. Independent ethics committees and international review boards provided written approval for the study protocol and all amendments. The ADAPT study was sponsored by argenx (Ghent, Belgium).

### Patient population

Inclusion criteria included participants ≥ 18 years of age with a diagnosis of MG with generalized muscle weakness, diagnosed as Myasthenia Gravis Foundation of America class II to IV, with an MG-ADL total score ≥ 5 points (> 50% of score attributed to nonocular symptoms), and on a stable dose of ≥ 1 gMG treatment prior to screening and throughout the study [22]. Acceptable concomitant treatments were limited to AChE inhibitors, corticosteroids, and NSISTs. The focus of this manuscript is the AChR-Ab+ subgroup, which represents a more-homogenous population. Additional details on inclusion and exclusion criteria for the ADAPT study were reported previously [22].

### HRQoL measures

The MG-QOL15r and EQ-5D-5L used to assess HRQoL in the ADAPT study were administered at initiation of each TC, weekly (± 1 day) during each TC, weekly (± 1 day) for 4 weeks after completion of each TC, then every 2 weeks, for ≤ 26 weeks.

The MG-QOL15r is a patient-reported disease-specific measure. The 15-item survey assesses patient perception of attributes associated with MG and emotional/psychological burden of MG, including extent of and dissatisfaction with MG-related dysfunction over the prior few weeks. Survey items are rated on a 3-point Likert scale of 0 to 2, where 0 is “not at all” and 2 is “very much.” There are 4 domains, with a differing number of items per domain: mobility (9 items), disease symptoms (3 items), general contentment (1 item), and emotional well-being (2 items). The 15 questions cover ocular, swallowing, and speech symptoms; proximal limb function and mobility; as well as personal grooming, social life and activities, disease fluctuations, and psychological health [23]. Higher scores indicate worse QoL. The measure has high reliability and has shown construct validity [23].

The EQ-5D-5L instrument developed by the EuroQoL Group is a standardized measure of health status widely used across disease states for clinical and economic appraisal. Patient-reported scores indicate perceived difficulty on that day for aspects of health across 5 dimensions: mobility, self-care, usual activities, pain/discomfort, and anxiety/depression. The 5 levels range from no problem (1) to unable to/severe problems (5). Scoring results in a 5-digit code that corresponds to the patient’s rating for each domain; this unique 5-digit code thus describes the patient’s health state [24]. The EQ-5D-5L health state can then be used to derive a utility score, also called the index value [24]. The utility score is derived by applying a formula of weighted values to an individual 5-digit score, then deducting the appropriate weights from the value for full health (i.e., 11111 = no reported problems in any of the 5 dimensions). Health utility scores generally range from 0 (equivalent to death) to 1 (equivalent to perfect health); higher utility scores reflect higher QoL [25]. The weights applied to calculate the utility score are standardized value sets based on representative samples from specific countries/regions. The UK value set was used to derive utility scores reported for ADAPT. A utility score can be used to calculate quality-adjusted life years and inform economic evaluations of health care interventions [24].

The EQ-5D-5L also includes a VAS that indicates a patient’s perceived overall health status for that day. Patients indicate their overall health on a scale of 0 to 100 by marking an *X* on a vertical scale of 5-digit increments, where 0 is “the worst health you can imagine” and 100 is “the best health you can imagine.” The numerical value where the *X* is placed is the VAS score [24]. Higher scores on the EQ-5D-5L VAS indicate better perceived overall health.

### Statistical analysis

HRQoL analyses in ADAPT were performed on the modified intent-to-treat (mITT) population, which included all randomly assigned participants with a value for the MG-ADL total score at baseline and at least 1 postbaseline timepoint. The mITT data set for the AChR-Ab+ group comprised all 129 randomly assigned AChR-Ab+ participants. Mixed-model repeated-measures analysis was used to compare data from HRQoL outcome measure scores, with least squares mean (LSM) difference and *P* values calculated for each visit. Effect sizes were calculated using Cohen’s *d* (difference between groups divided by pooled standard deviation). Effect sizes > 0.8 are considered a large effect [26]. Pearson correlation coefficients were computed to assess the linear relationship between MG-QOL15r scores and MG-ADL, EQ-5D-5L VAS, and EQ-5D-5L utility scores.

## Results

Of the 167 participants enrolled, 129 (77.2%) were AChR-Ab+ ; 38 participants (22.8%) were acetylcholine receptor antibody–negative (AchR-Ab–), of whom 6 (3.6%) were muscle-specific kinase–antibody–positive. In the AChR-Ab+ group, 65 were randomly assigned to efgartigimod and 64 to placebo. Patient demographics and baseline disease characteristics, disease status, and HRQoL assessment scores for the AChR-Ab+ participants are in Table 1. There were no notable differences between efgartigimod and placebo groups at baseline. The full listing of actual values for all HRQoL measures is included as Online Resource 1. Additional study baseline details can be found in the Howard et al. primary ADAPT study article [22].

**Table 1:** Demographics and baseline disease characteristics for AChR-Ab+ participants

	AChR-Ab+		
	Efgartigimod (*n* = 65)	Placebo (*n* = 64)	Total (*N* = 129)
Age, years; mean (SD)	44.7 (14.97)	49.2 (15.54)	46.9 (15.36)
Age category, years; *n*			
18 to ≤ 65	57	51	108
≥ 65	8	13	21
Sex at birth; *n*			
Female	46	40	86
Male	19	24	43
Time since diagnosis, years; mean (SD)	9.68 (8.25)	8.93 (8.21)	9.30 (8.21)
MG-ADL total score; mean (SD)	9.0 (2.48)	8.6 (2.14)	8.8 (2.32)
QMG total score; mean (SD)	16.0 (5.14)	15.2 (4.39)	15.6 (4.78)
MG-QOL15r; mean (SD)	15.7 (6.26)	16.6 (5.46)	16.2 (5.87)
Concomitant gMG treatment; *n*			
NSIST	40	37	77
Steroid	46	51	97
AChE inhibitor	57	57	114

*AChE* anticholinesterase, *AChR-Ab+ *acetylcholine receptor antibody–positive, *gMG* generalized myasthenia gravis, *MG-ADL* Myasthenia Gravis Activities of Daily Living, *n* number of participants for whom the observation was reported, *MG-QOL15r* Myasthenia Gravis-Quality of Life 15-item revised, *NSIST* nonsteroidal immunosuppressive therapy, *SD* standard deviation, *QMG* quantitative myasthenia gravis

Ranges for the clinical outcome assessments are as follows: MG-ADL total score 0 to 24, QMG score 0 to 39, and MG-QoL15r score 0 to 30; for each instrument, higher scores indicate more-severe disease

More information can be found in Howard JF, Jr., et al. Safety, efficacy, and tolerability of efgartigimod in patients with generalised myasthenia gravis (ADAPT): a multicentre, randomised, placebo-controlled, phase 3 trial. *Lancet Neurol*. 2021;20(7):526–536. 10.1016/S1474-4422(21)00159-9. Erratum in: *Lancet Neurol*. 2021;20(8):e5. PMID: 34146511

Significant improvements in HRQoL scores across multiple measurements were seen with efgartigimod compared to placebo. Even with poor overall HRQoL at baseline (as seen in Table 1), participants treated with efgartigimod showed rapid and substantial improvement in MG-QOL15r score and in all 5 dimensions of the EQ-5D-5L, as well as in the EQ-5D-5L VAS.

**Fig. 1:**
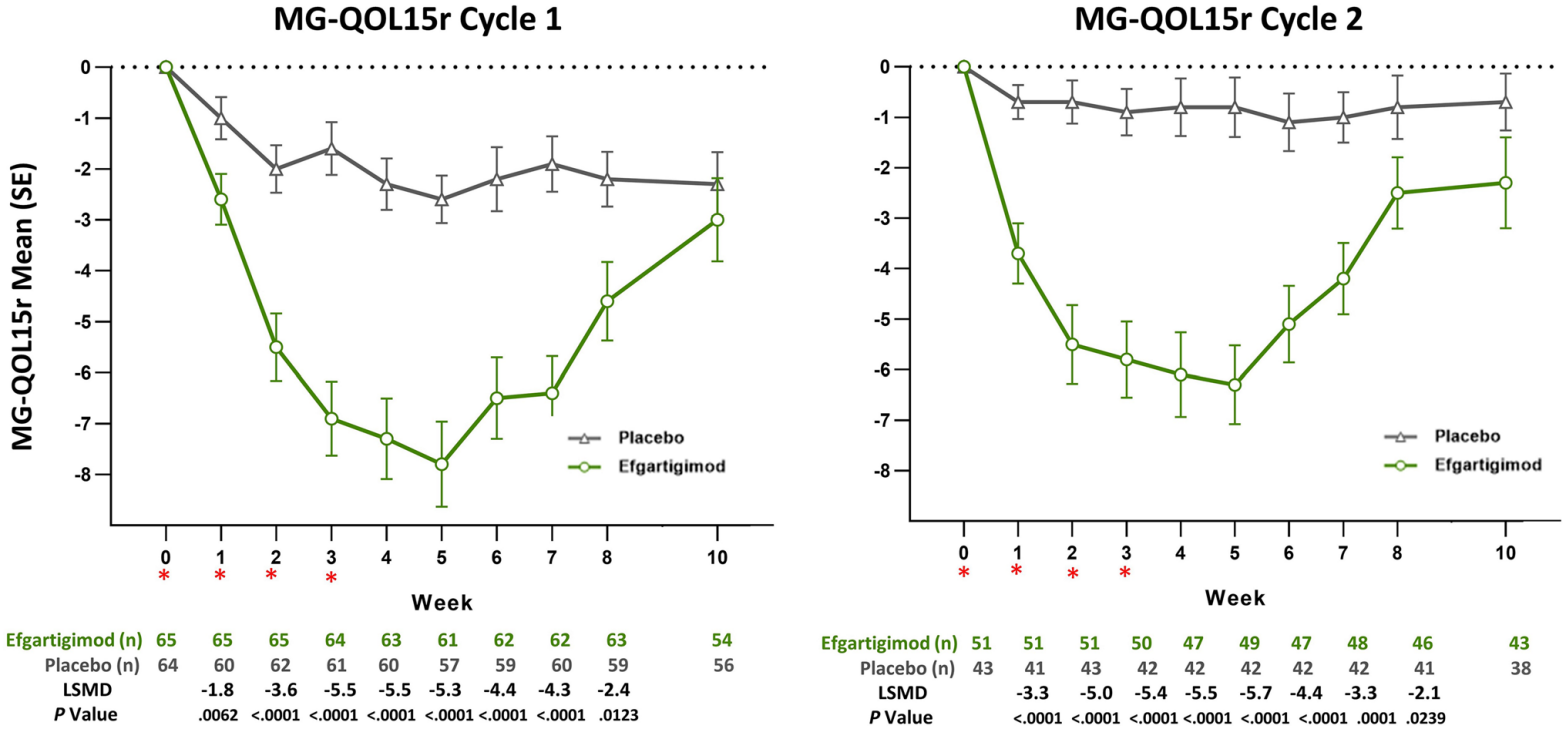
Mean change from baseline in MG-QOL15r score, by treatment cycle (AChR-Ab+ participants). *AChR-Ab+* acetylcholine receptor antibody receptor–positive, *LSMD* least squares mean difference, *MG-QOL15r* Myasthenia Gravis-Quality of Life 15-item revised, *SE* standard error. *indicates treatment administration (efgartigimod or matching placebo) timepoints (weeks 0, 1, 2, and 3)

**Fig. 2:**
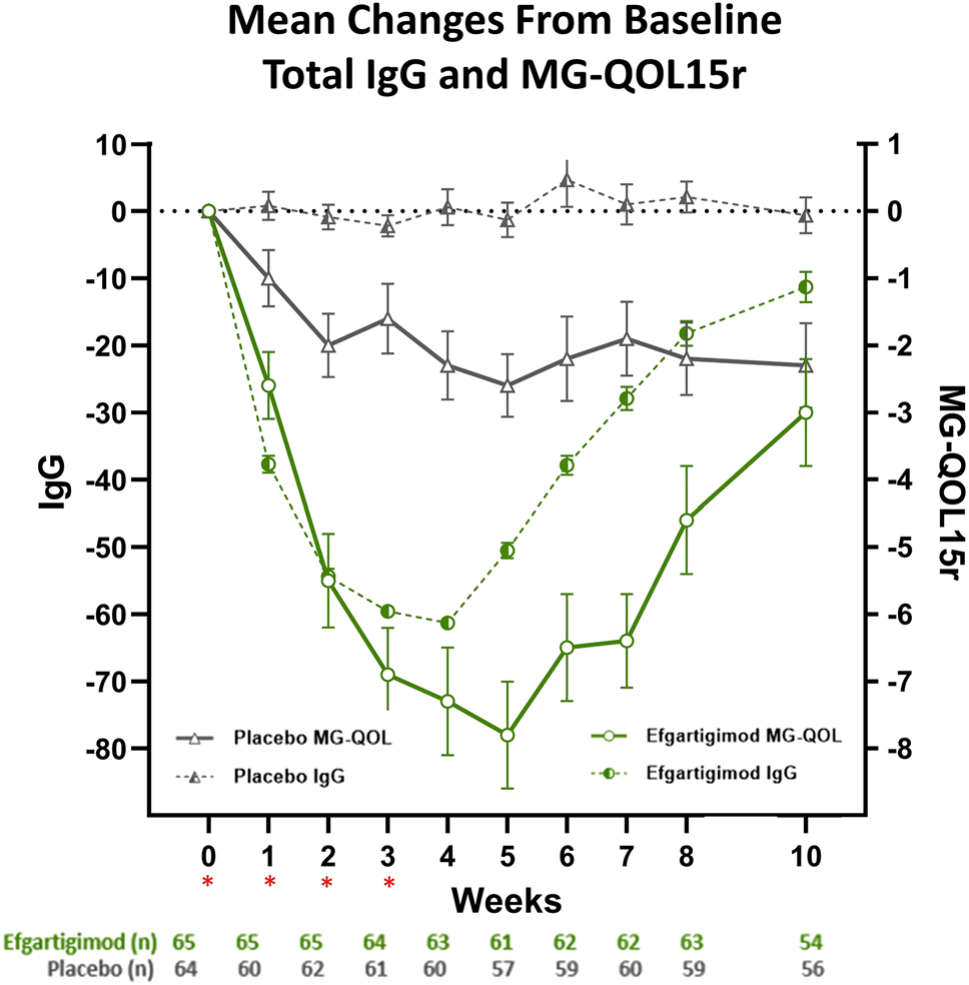
MG-QOL15r and total IgG for TC 1 in AChR-Ab+ participants. *AChR-Ab+* acetylcholine receptor antibody receptor–positive, *IgG* immunoglobulin G, *MG-QOL* Myasthenia Gravis-Quality of Life, *MG-QOL15r* Myasthenia Gravis-Quality of Life 15-item revised, *TC* treatment cycle. *indicates treatment administration (efgartigimod or matching placebo) timepoints (weeks 0, 1, 2, and 3)

There was a greater reduction in MG-QOL15r scores for AChR-Ab+ participants in the efgartigimod group vs participants in the placebo group (*P* < 0.0001); LSM changes from baseline, by week and TC, in MG-QOL15r score are shown in Fig. 1. Statistically significant differences were maintained for up to 8 weeks in TC 1 and TC 2. The largest between-group differences (> 5 points) were seen at weeks 3, 4, and 5 for efgartigimod-treated participants, and scores trended back toward baseline during the follow-up period of each TC. The MG-QOL15r score effect size at week 4 in TC 1 was 0.94. The trend in mean change in MG-QOL15r score was similar to the trend in mean change in total IgG level, by week, in TC 1 (Fig. 2).

**Fig. 3:**
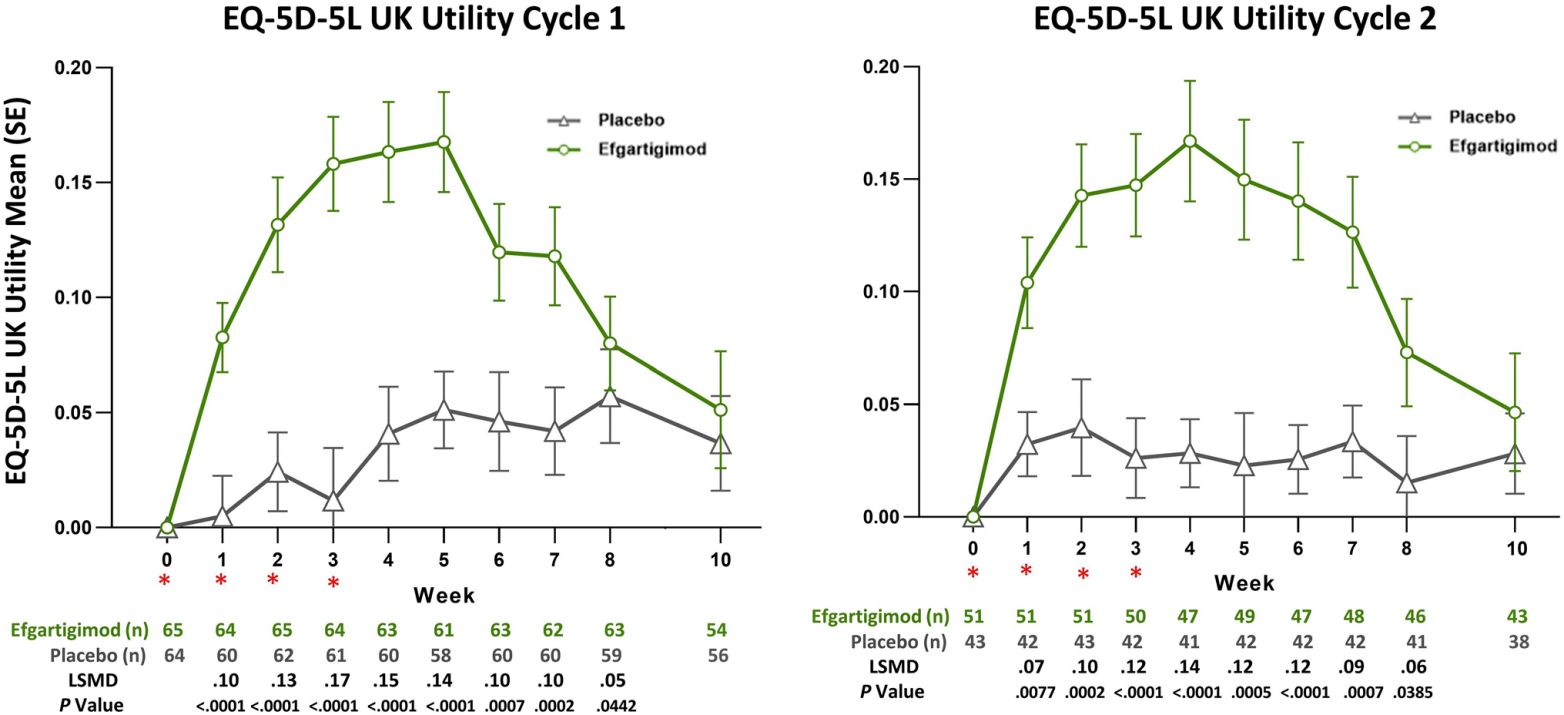
Mean change from baseline in EQ-5D-5L UK utility score, by treatment cycle (AChR-Ab+ participants). *AChR-Ab+* acetylcholine receptor antibody receptor–positive, *EQ-5D-5L* EuroQoL 5-Dimensions 5-Levels, *LSMD* least squares mean difference, *SE* standard error. *indicates treatment administration (efgartigimod or matching placebo) timepoints (weeks 0, 1, 2, and 3)

**Fig. 4:**
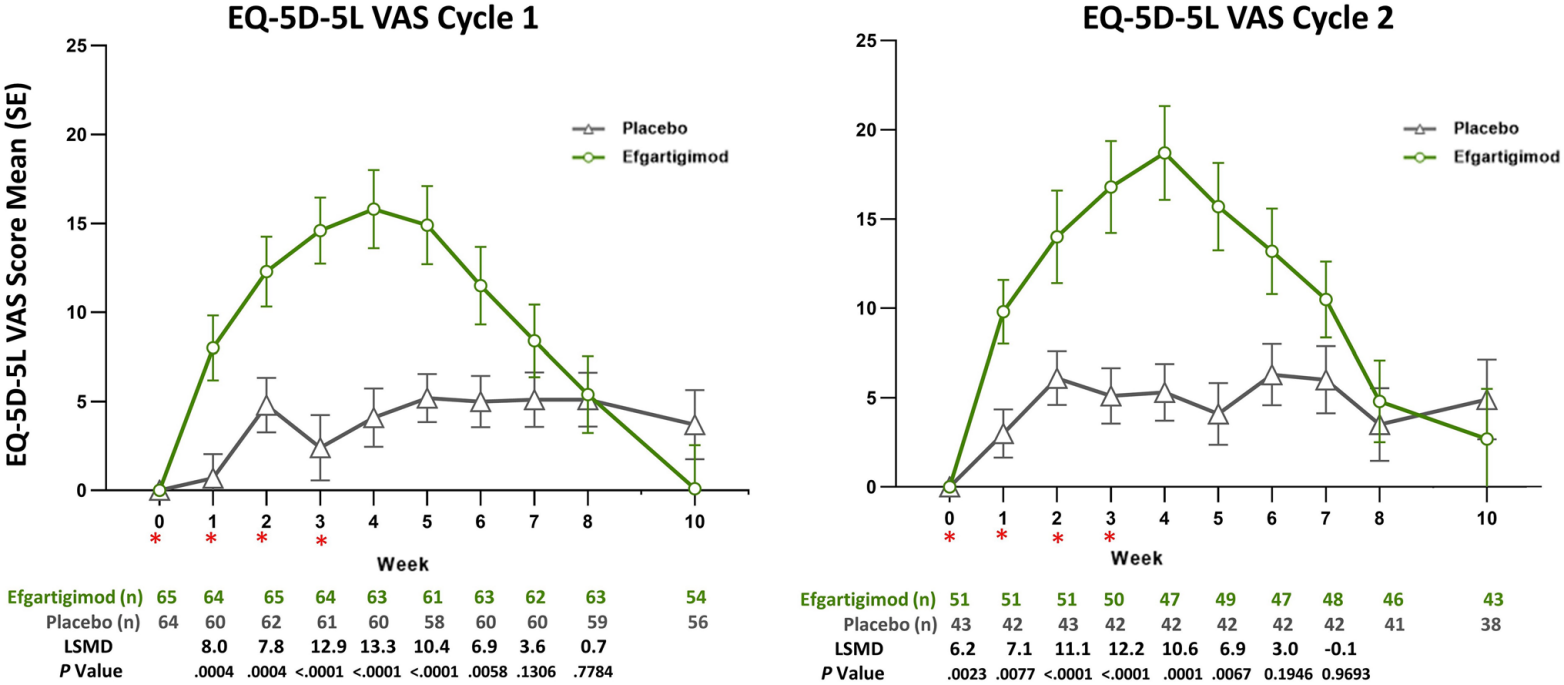
Mean change from baseline in EQ-5D-5L VAS score, by treatment cycle (AChR-Ab+ participants). *AChR-Ab+* acetylcholine receptor antibody receptor–positive, *EQ-5D-5L* EuroQoL 5-Dimensions 5-Levels, *LSMD* least squares mean difference, *SE* standard error, *VAS* visual analog scale. *indicates treatment administration (efgartigimod or matching placebo) timepoints (weeks 0, 1, 2, and 3)

**Fig. 5:**
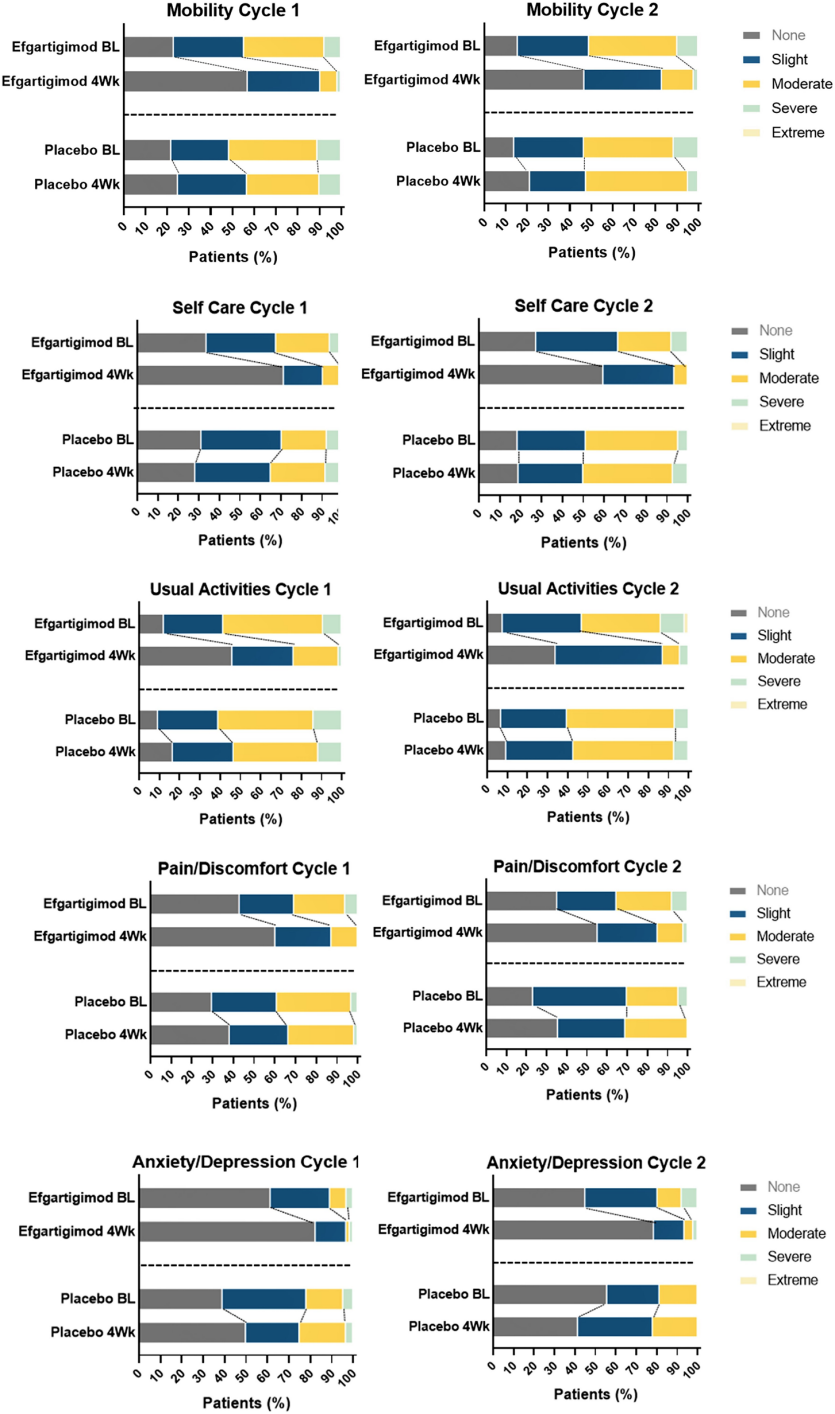
EQ-5D-5L domain responses, by treatment cycle (AChR-Ab+ participants). *4Wk* week 4, *AChR-Ab+* acetylcholine receptor antibody receptor–positive; *BL* baseline, *EQ-5D-5L* EuroQoL 5-Dimensions 5-Levels. Efgartigimod BL (*n* = 65), efgartigimod 4Wk (*n* = 63), placebo BL (*n* = 64), placebo 4Wk (*n* = 60)

There were also statistically significant differences in EQ-5D-5L utility and VAS scores between the efgartigimod and placebo groups, as measured by LSM change from baseline, by week, in TC 1 and TC 2 (Figs. 3 and 4). There were statistically significant differences in EQ-5D-5L utility scores in TC 1 and TC 2, which persisted up to 8 weeks. VAS scores were also statistically significantly different between the efgartigimod and placebo groups for weeks 1 through 5 in both TC 1 and TC 2. For each dimension of the EQ-5D-5L, participants treated with efgartigimod showed improvement, whereas participants treated with placebo did not (Fig. 5). For mobility, the percentage increase in participants reporting no problems (averaged over TC 1 and TC 2) was 38% for the efgartigimod group vs 7% for placebo. For self-care, the averaged percentage increase in participants reporting no problems was 36% for the efgartigimod group vs an averaged decrease of 1.5% for the placebo group. For usual activities, the efgartigimod group had an averaged increase in reporting no problems of 30% vs 9.5% for placebo; for pain/discomfort, increase for efgartigimod was 19% vs 11% for placebo; and for anxiety/depression, efgartigimod was an increase of 13% vs a decrease of 1.5% for the placebo group.

**Fig. 6:**
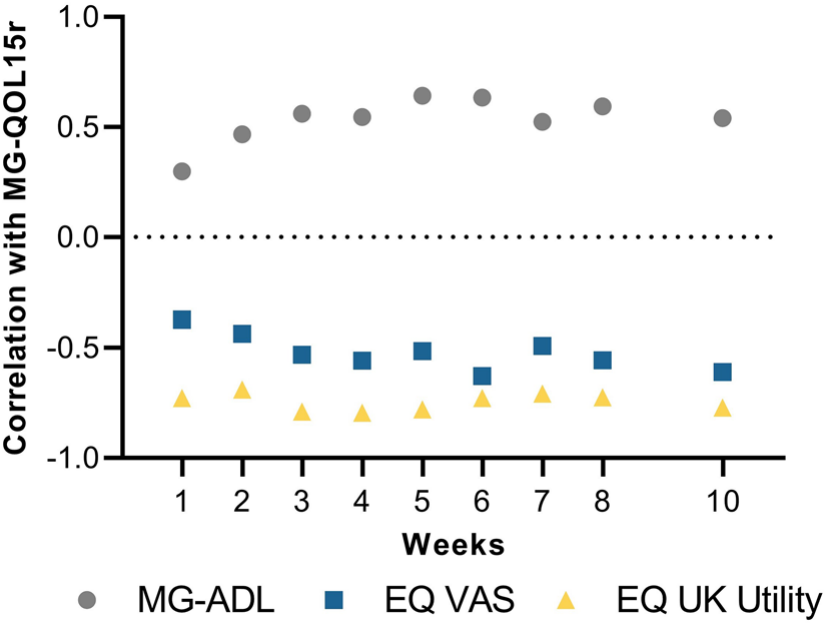
Correlations between MG-QOL15r scores and other assessment scores for efgartigimod group (TC 1; AChR-Ab+ population). *AChR-Ab+* acetylcholine receptor antibody receptor–positive, *EQ-5D-5L* EuroQol 5-Dimensions 5-Levels, *EQ VAS* EuroQol visual analog scale, *MG-ADL* Myasthenia Gravis Activities of Daily Living, *MG-QOL15r* Myasthenia Gravis Quality of Life 15-item revised, *TC* treatment cycle. Lower scores equate to better outcomes for both MG-QOL15r and the MG-ADL; higher scores equate to better outcomes for both the EQ-5D-5L VAS and utility values

There were statistically significant correlations between MG-QOL15r and EQ-5D-5L VAS and utility scores for the efgartigimod group (Fig. 6). A positive correlation was shown between MG-QOL15r and MG-ADL total scores over the first TC (week 1, *P* = 0.0157; weeks 2–10, *P* < 0.0001); significant negative correlations were shown between MG-QOL15r and both the EQ-5D-5L VAS scores (week 1, *P* = 0.0024; week 2, *P* = 0.0003; weeks 3–10, *P* < 0.0001) and EQ-5D-5L UK utility scores (weeks 1–10, *P* < 0.0001). The strongest correlation was seen between MG-QOL15r and the EQ-5D-5L utility scores.

## Discussion

Efgartigimod had a rapid impact on HRQoL, with significant improvements as early as the first week of treatment in each TC. These improvements were consistent across multiple measures and were similar for the effect seen in TC 1 and TC 2. The observed benefits on HRQoL were reproducible and durable.

At baseline and despite being on stable treatment at screening, participants in this study had fairly low EQ-5D-5L health utility scores (0.62–0.66), highlighting the potential burden of gMG. The majority reported issues with mobility, usual activities, and self-care and a considerable proportion reported pain/discomfort and anxiety/depression. These baseline levels underscore the need for more-effective treatment options for this patient population.

The study did not include formal data analysis of AChR-Ab− participants due to the small sample size; the study was not powered to demonstrate statistically significant differences between the efgartigimod and placebo groups in the AChR-Ab− population. However, results for MG-QOL15r and EQ-5D-5L were similar for the overall efgartigimod group (AChR-Ab+ and AChR-Ab−), indicating that there were no substantial differences between the AChR-Ab+ and AChR-Ab− populations.

Substantial and consistent improvements were seen in disease-specific MG-QOL15r scores. The MG-QOL15r apprises a clinician of how a patient has evaluated their level of satisfaction/dissatisfaction with current MG manifestations and offers an efficient and standardized assessment of physical, social, and psychological domains that is specific to patients with MG [27, 28]. As such, it is a good indicator of disease status and disability, although the minimally important difference has not yet been established [29].

Improvements were seen across all dimensions captured in the EQ-5D-5L for the efgartigimod group compared with little or no improvement for the placebo group. The maximum mean difference from TC baseline in EQ-5D-5L utility score was seen at 4 weeks in both TC 1 and TC 2 for the efgartigimod group (TC 1, 0.158; TC 2, 0.187) and was higher than the estimated minimally important difference cut point for the EQ-5D-5L utility scores (range: 0.03–0.05) [30]. Maximum mean difference in utility score for the placebo group was 0.052 at week 5 in TC 1 and 0.063 at week 6 in TC 2.

The pattern of improvements in HRQoL corresponded well with the change in IgG level, as shown in Fig. 2, and was consistent with patterns observed for other efficacy measures. Maximum benefit was observed at week 4 or 5 in each TC, similar to the timepoint with greatest change in IgG level. This pattern is consistent with similar trends seen between symptom improvement and reductions in IgG level reported previously [22]. Interestingly, the onset of the beneficial clinical effect paralleled the decrease in IgG titer, but the return of clinical myasthenic symptoms showed a delay of about 3 weeks compared to the rise in IgG titer. The ADAPT study design was well suited to evaluate the repeatability of the treatment effect, and we saw a similar magnitude of benefit on HRQoL with efgartigimod in TC 1 and TC 2. The patterns of variation in trends and timing of effects in HRQoL measures were consistent with other clinical measures in the ADAPT study.

We evaluated whether the benefits of efgartigimod were consistent among key patient demographic characteristics known to impact HRQoL and found consistent treatment benefit. Certain patient demographic criteria, including female sex and increasing age, have been identified as drivers of HRQoL in MG [5, 10, 13, 31]. Due to the limited sample size and the RCT setting, the ADAPT study was not the ideal dataset to evaluate drivers, adjusted by key patient demographic characteristics known to impact HRQoL, and corresponding interaction with treatment found consistent treatment benefit.

Strong correlations have been reported between symptom control and HRQoL benefit [27, 32, 33]. The findings from this study show HRQoL results that are strongly tied to symptom improvement scores (ie, MG-ADL). The results demonstrate efgartigimod’s effect on clinical symptoms [22] and HRQoL; however, clinical improvement without significant improvement in HRQoL has also been seen in studies of other MG therapeutics [16, 34]. Clinical manifestations of MG may not fully portray the burden of MG [35] and, conversely, there may be additional therapeutic benefit that is unaccounted for by measuring symptom control alone.

Given such findings, HRQoL measures are becoming increasingly important for evaluating the benefit of therapeutic interventions. They have historically been undervalued both in clinical studies of gMG and in clinical practice, where treatment intent has been primarily focused on preventing hospitalization. These measures, however, better assess the full burden of disease experienced by individuals with gMG; HRQoL measures reflect patients’ perspective on the effectiveness of treatment and disease burden and can be used to foster patient-centric care in clinical practice. Regulatory bodies have also recognized the importance of patient-reported outcome measures, including HRQoL, and are increasingly using these data to support label claims and reimbursement decisions. The consistent and substantial achievement of clinical and HRQoL end points in this study demonstrates the broader benefit of treatment with efgartigimod beyond relief of immediate signs and symptoms of gMG.

A limitation of this study is that although fatigue is an important aspect of gMG, it is not fully captured by the assessment tools utilized. This might explain the difference seen between the EQ-5D-5L VAS scores, which represent participants’ perceived overall health, and EQ-5D-5L utility scores, which represent perceived problems in specific dimensions. Findings were based on clinical study data with limited data/follow-up and a specific dosing schedule. The study design evaluated duration of effect after each TC, which did not allow extrapolation of HRQoL impact to long-term real-life scenarios wherein patients are treated according to neurologic evaluation. Future studies using real-world data will be needed to further validate the benefit on HRQoL of efgartigimod in gMG.

## Conclusion

Treatment with efgartigimod resulted in significant and rapid HRQoL improvements for up to 8 weeks after the first infusion in TC 1 and TC 2.

## Supplementary Information

Below is the link to the electronic supplementary material.

Supplementary file1 (PDF 135 KB)

## Data Availability

argenx is committed to responsible data sharing regarding the clinical trials they fund. Included in this commitment is access to anonymized, individual, and trial-level data (analysis datasets), and other information (e.g, protocols and clinical study reports), as long as the trials are not part of an ongoing or planned regulatory submission. This includes requests for clinical trial data for unlicensed products and indications. These clinical trial data can be requested by qualified researchers who engage in rigorous independent scientific research and will only be provided after review and approval of a research proposal and statistical analysis plan and execution of a data sharing agreement. Data requests can be submitted at any time and the data will be accessible for 12 months. Requests can be submitted to ESR@argenx.com.
