# Supplementary material for: Efgartigimod improved health-related quality of life in generalized myasthenia gravis: results from a randomized, double-blind, placebo-controlled, phase 3 study (ADAPT)
Source: J Neurol. 2023 Jan 4;270(4):2096–105. doi: 10.1007/s00415-022-11517-w (PMC10025199; doi:10.1007/s00415-022-11517-w)
Supplement: Supplementary file 1 — Supplementary file1 (PDF 135 KB) [file 415_2022_11517_MOESM1_ESM.pdf]

Online Resource 1: Actual Values for MG-QOL15r, EQ-5D-5L UK Utility, and EQ-5D-5L VAS Scores (AChR-Ab+ Patients)

	Efgartigimod (n=65)		Placebo (n = 64)	
	**Mean (SE)**	**n**	**Mean (SE)**	**n**
**MG-QOL15r**				
Cycle 1				
Week 1	13.1 (0.78)	65	15.7 (0.79)	60
Week 2	10.2 (0.79)	65	14.7 (0.71)	62
Week 3	8.7 (0.74)	64	15.1 (0.81)	61
Week 4	8.3 (0.82)	63	14.4 (0.78)	60
Week 5	7.8 (0.77)	61	14.1 (0.78)	57
Week 6	9.3 (0.89)	62	14.3 (0.84)	59
Week 7	9.4 (0.80)	62	14.7 (0.76)	60
Week 8	10.9 (0.87)	63	14.5 (0.79)	59
Week 10	12.8 (0.99)	54	13.9 (0.81)	56
Week 12	10.2 (1.36)	26	11.7 (1.89)	16
Week 14	10.6 (2.04)	16	9.0 (1.34)	16
Week 16	9.2 (2.03)	14	9.4 (1.45)	15
Week 18	7.7 (2.39)	12	8.7 (1.42)	13
Week 20	3.9 (1.34)	9	9.7 (1.56)	11
Week 22	3.7 (2.08)	6	9.5 (1.74)	11
Week 24	1.6 (1.17)	5	10.7 (1.96)	9
Week 26	0.5 (0.50)	4	11.6 (1.70)	7
Cycle 2				
Week 1	11.6 (0.85)	51	15.0 (0.82)	41
Week 2	9.8 (0.90)	51	15.0 (0.85)	43
Week 3	9.5 (0.91)	50	15.0 (0.85)	42
Week 4	9.3 (1.00)	47	15.0 (0.77)	42
Week 5	9.0 (0.95)	49	14.8 (0.95)	42
Week 6	10.4 (0.90)	47	14.9 (0.87)	42
Week 7	11.3 (0.96)	48	14.7 (0.83)	42
Week 8	12.4 (0.95)	46	15.0 (0.94)	41
Week 10	13.1 (0.91)	43	14.6 (0.90)	38
Week 12	10.1 (2.92)	7	13.4 (2.07)	7
**EQ-5D-5L Utility**				
Cycle 1				
Week 1	0.7424 (0.0190)	64	0.6122 (0.0228)	60
Week 2	0.7911 (0.0203)	65	0.6361 (0.0210)	62
Week 3	0.8184 (0.0185)	64	0.6229 (0.0260)	61
Week 4	0.8209 (0.0194)	63	0.6511 (0.0237)	60
Week 5	0.8237 (0.0199)	61	0.6580 (0.0195)	58
Week 6	0.7743 (0.0222)	63	0.6565 (0.0219)	60
Week 7	0.7777 (0.0208)	62	0.6524 (0.0207)	60
Week 8	0.7408 (0.0209)	63	0.6628 (0.0215)	59
Week 10	0.7096 (0.0244)	54	0.6549 (0.0207)	56
Week 12	0.7333 (0.0411)	26	0.6634 (0.0653)	16
Week 14	0.6992 (0.0631)	16	0.7514 (0.0411)	16
Week 16	0.7639 (0.0708)	14	0.7254 (0.0618)	15
Week 18	0.8256 (0.0425)	12	0.7835 (0.0474)	13

Week 20	0.8946 (0.0457)	9	0.7664 (0.0473)	11
Week 22	0.9405 (0.0378)	6	0.7540 (0.0473)	11
Week 24	0.9432 (0.0354)	5	0.7063 (0.0609)	9
Week 26	0.9083 (0.0536)	4	0.7183 (0.0652)	7
Cycle 2				
Week 1	0.7128 (0.0296)	51	0.6533 (0.0219)	42
Week 2	0.7516 (0.0284)	51	0.6599 (0.0187)	43
Week 3	0.7566 (0.0235)	50	0.6472 (0.0241)	42
Week 4	0.7767 (0.0254)	47	0.6418 (0.0218)	41
Week 5	0.7595 (0.0230)	49	0.6415 (0.0311)	42
Week 6	0.7574 (0.0241)	47	0.6423 (0.0213)	42
Week 7	0.7290 (0.0247)	48	0.6509 (0.0207)	42
Week 8	0.6778 (0.0235)	46	0.6397 (0.0230)	41
Week 10	0.6611 (0.0244)	43	0.6519 (0.0198)	38
Week 12	0.7277 (0.0884)	7	0.6807 (0.0444)	7
**EQ-5D-5L VAS**				
Cycle 1				
Week 1	66.3 (2.13)	64	57.4 (2.58)	60
Week 2	70.5 (2.09)	65	61.3 (2.16)	62
Week 3	73.2 (2.13)	64	58.7 (2.47)	61
Week 4	74.4 (2.07)	63	60.6 (2.28)	60
Week 5	74.0 (2.02)	61	61.4 (2.42)	58
Week 6	69.5 (2.26)	63	61.5 (2.41)	60
Week 7	67.0 (2.23)	62	61.6 (2.28)	60
Week 8	63.6 (2.42)	63	61.7 (2.27)	59
Week 10	60.0 (2.85)	54	59.7 (2.36)	56
Week 12	71.2 (3.36)	26	62.8 (6.45)	16
Week 14	70.6 (4.94)	16	68.3 (3.85)	16
Week 16	74.4 (4.74)	14	64.9 (5.27)	15
Week 18	77.5 (3.96)	12	70.2 (4.77)	13
Week 20	81.0 (4.30)	9	69.4 (5.13)	11
Week 22	88.5 (4.08)	6	71.6 (5.07)	11
Week 24	88.0 (4.90)	5	62.2 (6.30)	9
Week 26	92.8 (2.59)	4	66.4 (6.24)	7
Cycle 2				
Week 1	62.7 (2.16)	51	59.1 (2.75)	42
Week 2	66.9 (2.27)	51	62.5 (2.77)	43
Week 3	69.7 (2.13)	50	61.2 (2.96)	42
Week 4	71.2 (2.27)	47	61.6 (2.90)	42
Week 5	69.1 (2.29)	49	60.9 (3.02)	42
Week 6	66.4 (2.28)	47	62.1 (2.98)	42
Week 7	62.2 (2.18)	48	62.8 (2.82)	42
Week 8	57.7 (2.23)	46	60.4 (3.13)	41
Week 10	54.9 (2.49)	43	63.0 (2.76)	38
Week 12	59.6 (10.53)	7	69.0 (7.77)	7

AChR-Ab+ acetylcholine receptor antibody receptor–positive, *EQ-5D-5L*, EuroQoL 5-Dimensions 5-Levels, *MG-QOL15r* Myasthenia Gravis-Quality of Life 15-item revised, *SE* standard error, *VAS* visual analog scale
